# ANN based surrogate model for key Physico-chemical effects of cavitation

**DOI:** 10.1016/j.ultsonch.2023.106327

**Published:** 2023-02-11

**Authors:** Nanda V. Ranade, Vivek V. Ranade

**Affiliations:** a 47 Halcyon Place, Castletroy, Limerick, Ireland; b Bernal Institute, University of Limerick, Limerick, Ireland

**Keywords:** Hydroxyl radicals, Jet velocity, Localised energy dissipation, Surrogate model

## Abstract

Intense and localised physico-chemical effects realised by cavitation such as generation of hydroxyl radicals, high-speed jets, and very high energy dissipation rates are being harnessed for a wide range of applications from emulsions, crystallisation, reactions to water treatment and waste valorisation. Single cavity models are typically used to quantitatively estimate such localised effects of cavity collapse. However, these models demand significant computing resources for resolving fast dynamics and therefore are very difficult, if not impossible, to integrate with CFD based cavitation device or reactor scale models. This severely limits the utility of device/ reactor scale models in simulating key applications of interest. In this work, we present, for the first time, artificial neural network (ANN) based surrogate models which accurately represent complex physico-chemical effects of cavity collapse. Recently developed cavity dynamics model was used for generating training data set encompassing both acoustic and hydrodynamic cavitation. Appropriate methodology for training ANN was developed. A shallow three hidden layer dense ANN was found to be more effective for estimating three main effects of cavity collapse: jet velocity, •OH generation and localised energy dissipation rate. The performance of trained ANN was then evaluated by comparing the predictions with the totally unseen data obtained from the cavity dynamics model. The developed ANN was shown to simulate unseen data very well not just within the range of training data (interpolation) but also beyond (extrapolation). Algebraic equations representing ANN are included to facilitate incorporation in device/ reactor scale CFD models. The presented methodology and results will be useful for developing high-fidelity CFD models of cavitation devices/ reactors based on key physico-chemical effects of cavity collapse.

## Highlights

• Developed shallow multilayer dense ANN model to simulate physico-chemical effects of cavity collapse.
• The ANN model showed excellent performance for interpolation as well as extraploation.
• The developed algebraic model equations are useful for incorporating in high fidelity CFD models of cavitation devices.

## 1 Introduction

Cavitation is a phenomenon of formation, growth and implosion of vapour filled cavities. The implosion of cavity leads to very high pressures and temperatures, high velocity jets, and intense localised energy dissipation rates and strongly oxidising hydroxyl radicals (Shah et al. [1], Brennan [2], Ranade et al. [3]). These extreme physio-chemical effects produced by cavitation are of interest to engineers and researchers primarily for two reasons (a) to estimate and possibly avoid damage of equipment and (b) to harness these extreme effects for beneficial applications. For both these areas of interest, it is crucial to develop appropriate models for estimating intense physico-chemical effects of cavitation. Several attempts have been done for developing such models which may be broadly classified into (a) simplified one-dimensional (1D) models based on Rayleigh-Plasset equations or their variants (see for example, Pandit et al. [4]) and (b) Multi-dimensional CFD based models (see for example, Orthaber et al. [5] using volume of fluid approach or Shan et al. [6] using lattice Boltzmann approach). In recent years, several attempts of developing multi-scale models of cavitating flows have been made (see for example, [7], [8], [9]). However, the focus in such multi-scale models is on simulating overall flow characteristics rather than capturing details of cavity collapse. Considering the associated uncertainties in estimating physico-chemical properties at extreme pressures and temperatures generated during cavity collapse, 1D models of cavity collapse based on Rayleigh-Plasset type of equations appear to be a pragmatic choice at the moment.

Recently Pandit et al. [4] have critically reviewed different variants of Rayleigh-Plasset type of equations used for simulating cavity dynamics and have presented simulated results on jet velocity, hammer pressure, hydroxyl radicals and local energy dissipation rates over the wide parameter space covering ambient temperature, pressure, amplitude and frequency of pressure fluctuations and initial cavity radius. These estimated physico-chemical effects of cavitation can be used for developing a device scale model for simulating performance of cavitation based reactor/ process (see recent perspective by Ranade [10]). However, incorporation of complex cavity dynamics model requiring very fine spatio-temporal resolution in the macroscopic model is not straight forward and is extremely compute intensive. Some attempts of developing empirical correlations based on the results of detailed cavity dynamic models (see for example, Gogate and Pandit [11] or Tao *et al*. [12]) have been made. Not unsurprisingly, considering strong nonlinearities and complex underlying physics of cavity collapse, applicability of regressed correlations was rather limited. ANNs are known to be capable of describing complex relationships [13] and are ideal platform for developing a surrogate model representing non-linear processes. The trained (fitted) ANN based surrogate model can be expressed in terms of algebraic equations requiring orders of magnitude less computations than the rigorous model and can therefore be incorporated conveniently in the macroscopic or device/ reactor scale models. In this work, we have developed an effective surrogate model which mimics complex cavity dynamics model over a wide range of parameters using artificial neural network (ANN).

**Fig. 1:**
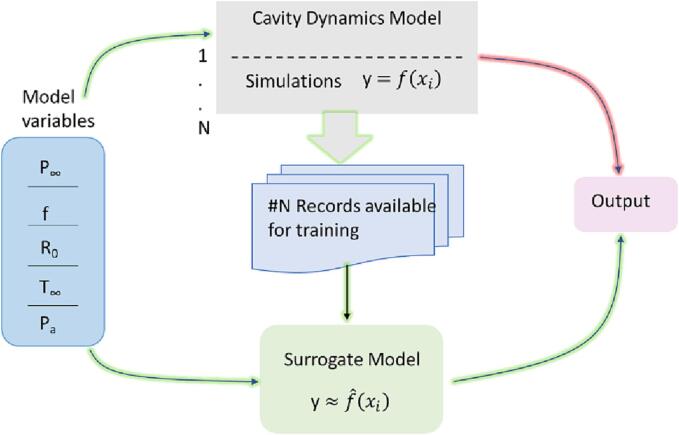
Approach used in this work.

We selected recently reported cavity dynamics model (CDM) by Pandit et al [4] for this purpose. Their model was used to simulate cavity dynamics over a wide range of parameters relevant to acoustic and hydrodynamic cavitation (ambient pressure, $P_{\infty }$; ambient temperature, $T_{\infty }$; frequency of pressure fluctuations, *f*; amplitude of pressure fluctuations $P_{a}$ and initial cavity radius, $R_{0}$) for estimating key physico-chemical effects of interest during cavity collapse (hammer pressure, jet velocity, number of OH radicals generated and localised energy dissipation rates). Using these simulated results, we developed ANN based surrogate model (see schematic of this approach in Fig. 1).

Part of the simulated results obtained from solution of CDM was used for training the ANN models. The performance of ANN models was tested with data not seen by ANN during the development, training and testing. The test data was divided into two groups, in order to evaluate performance for interpolation (within the range of training data) as well as extrapolation (beyond the range of training data). The approach and presented model/ model parameters will be very useful for incorporating the surrogate model of cavity dynamics into macroscopic models of cavitation devices/ reactors.

## 2 Data, architecture and training for ANN models

Features of available data and its pre-processing, the architecture and specifications of individual ANN models and the training process is discussed in this section.

### 2.1 Available data and architecture of ANN model

The data used in this work is taken from the work of Pandit et al [4]. They have simulated cavity dynamics by systematically varying five independent variables as indicated in Table 1. It should be noted that the cavity collapse and associated effects are relevant only beyond the Blake threshold where abrupt changes are observed (see Yasui et al. [14]). Any practical application of cavitation will operate away from the vicinity of the Blake threshold. Considering this Pandit et al. [4] have carried out simulations of cavity dynamics by considering parameters away from the Blake threshold. The range of simulated results is indicated in Table 1. Simulation results were available as 225 records with various combinations of the independent variables and the various key output variables of interest. In this work, jet velocity, hydroxyl radical generation and energy dissipation rate were selected as key physico-chemical effects of interest.

**Table 1:** Input variables and results from cavity dynamics simulations.

Input Variable	Values
Ambient pressure, $P_{\infty }$, Pa	14162.88, 53110.81, 101325, 212443.2
Ambient temperature, $T_{\infty }$, K	290, 300, 310, 320
Pressure amplitude, $P_{A}^{\prime }=P_{a}/P_{\infty }$	1, 1.2, 1.4, 1.6, 1.8, 2.0, 2.2, 2.4, 2.6, 2.8, 3.0
Initial cavity radius, $R_{0}$, *µm*	2, 5, 10, 20, 50
Frequency of pressure fluctuations, f, kHz	5, 10, 20, 40, 80
Output results	Range
Jet velocity ($V_{jet}$), m/s	10.16–194.77
•OH Generation ($n_{OH}$), number of radicals	31.73–8.47E + 12
Localized Energy Dissipation Rates (ε), m^2^/s^3^	40436.40 – 5.65E + 08

Since the available results were obtained through simulations, pre-processing for handling noise was not required. However, the range of generated hydroxyl radicals and localised energy dissipation rate spanned 11 and 4 orders of magnitude respectively. These two variables were used after taking their natural log while developing and training the ANN models. The output of trained models was post-processed with exponential function to get the final output.

In order to evaluate the interpolation and extrapolation performance of developed ANN model, it was decided to keep some of the available results (out of the 225) as unseen data and was not used for developing and training the ANN models. All the results obtained with $P_{A}^{\prime }$ value equal to 2 (23 records) were selected as an unseen data for evaluating interpolation performance since this value of $P_{A}^{\prime }$ falls between the remaining values of $P_{A}^{\prime }$. All the results with $P_{A}^{\prime }$ equal to 3 (24 records) were selected as an unseen data for evaluating extrapolation performance of the trained network as this value of $P_{A}^{\prime }$ falls outside the remaining values of $P_{A}^{\prime }$. Checking the performance for variables outside the training range is particularly important for ANN models. This is because if the model shows good performance for extrapolation, it is an indication that it has successfully captured the underlying physical phenomena. The remaining 178 records (225–23-24) were used for training and development of ANN models.

MATLAB R2022a platform was used to construct the desired ANN model. Specifically, the ‘network’ function available for building custom shallow neural networks was used. It is always important to think through the inputs and desired outputs of ANN model before developing the internal architecture of the ANN model. The available data indicated dependency of three output variables on five input variables. There were two ways to implement this using ANN. One was to build a single ANN with five inputs and three outputs whereas the other was to build three different ANNs each with five inputs and one output. Though a single ANN giving all the three variables of interests looks attractive, it was not opted for three reasons. Firstly, single ANN with three outputs would have higher number of parameters to be decided than individual ANNs with single output. Provided that limited number of records were available for training, efforts were directed to minimize the number of parameters to be fitted. Secondly, for a particular application of cavitation, users might not be interested in all three variables simultaneously. For example, application involving chemical effects of cavitation would need to consider •OH generation; whereas that involving physical effects would need to take into account energy dissipation rate or jet velocity instead. Thirdly, a close look at cavity dynamics model used as a reference in this work indicates that the range of chosen output variables as well as their dependency with respect to input variables is quite different from each other. Instead of making a single network to adjust its parameters for such intrinsically differing outputs, it was decided to develop separate function fitting ANN models for each of the output variables. This allowed the use of all available records for training each model. Thus, every model had five variables in Table 1 as input and one of the result variables as output.

The architecture of any ANN is defined by number of hidden layers, the neurons in each layer and their interconnections. Universality theorem states that neural networks with a single hidden layer can be used to approximate any continuous function to any desired precision [15]. Hence the natural choice to start with was to have a single hidden layer in ANN model. However, it is also shown that there is a simple (approximately radial) function expressible by a small 3-layer feedforward neural networks, which cannot be approximated by any 2-layer network, to more than a certain constant accuracy, unless its width is exponential in the dimension [16]. This formally demonstrates that depth – even if increased by 1 – can be exponentially more valuable than width (number of neurons) for standard feedforward neural networks. In light of this, it was decided to start the development with a single hidden layer. If the quality of estimates found to be below acceptable level, either the number of neurons or number of hidden layers were increased. Before starting the development of ANN, it is important to identify limiting values of number of neurons/ layers considered in the model with reference to the number of available training records, $N_{\mathrm{Train}}$. There are unfortunately no fixed rules for identifying such limiting values. Intuitive reasoning and thumb rules are generally used to assist the decision. In neural networks, weights and biases are the unknown parameters. ‘Rule of 10′ says that required number of records to train is 10 times the total number of parameters in the model [18]. However, following this rule of 10 may restrict number of potential neurons used in the ANN model to very small number. In this work, therefore, we kept the limit on maximum number of unknown parameters as half of total available training records. For a single hidden layer ANN with $n_{i}$ inputs and $n_{o}$ outputs, maximum number of neurons $H_{\max}$, can be given as:

(1) $$H_{\max}=\frac{N_{\mathrm{Train}}}{2\left[\left(n_{i}+1\right)H+n_{o}H\right]}$$

In case of more than one hidden layer, one needs to calculate the total number of parameters by considering weights and bias for individual layers following a similar logic. The thumb rules for deciding neurons in additional hidden layers are either use 0.5 times the neurons in previous layer or 2/3rd of addition of neurons in previous and next layer [19]. The purpose to introduce more hidden layers in present work was to increase model’s capacity to represent complex nonlinear functions using limited neurons. The basis for this choice was that the cascaded stages of hidden layers would combine the inputs using transfer function $f_{\mathrm{hi}}$ of each added layer ‘*i*’ in more complex way as given by Equation (2).

(2) $$\mathrm{Output}=f_{h3}\left(f_{h2}\left(f_{h1}\left(Inputvariables\right)\right)\right)$$

Hence, in this work, additional layers were used with limited neurons but having full connectivity (dense layers). This is in contrast with deep networks where additional layers may not have full connectivity; the objective there being looking for a particular feature. The total number of neurons to be used in multilayer models were chosen with an upper limit of $H_{\max}$.

Following this philosophy, we started the ANN model development with a single hidden layer ANN model with an arbitrarily small number of neurons in the hidden layer (<$H_{\max}$). For each case, 1000 different networks for a particular number of neurons in the hidden layer were trained. These 1000 networks differ in their initial weights and hence perform differently resulting in different values for R-squared (Ranade et al. [17]). The model with the highest R-squared value was selected. If this highest value of R-squared was found to be less than 0.995, number of neurons in the hidden layer was incremented by one till either the best R-squared value for training set is >= 0.995 or number of neurons becomes $H_{\max}$. If desired value of R-squared is not reached despite using maximum permissible number of neurons, multiple hidden layer models were used.

### 2.2 Training

**Fig. 2:**
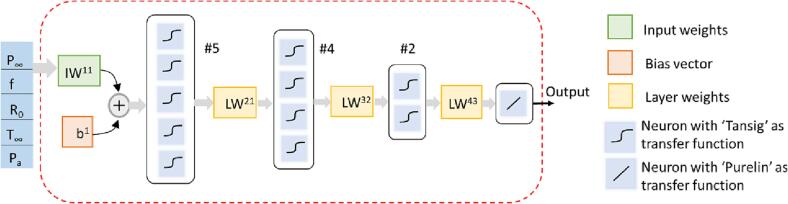
Architecture of selected multilayer ANN.

The developed networks were trained using MATLAB function ‘train’ which implements batch training for shallow networks. Initially-two training functions were considered, ‘trainlm’ and ‘trainbr’. Both the functions have back propagation algorithm which uses the Levenberg-Marquardt optimization. These two functions use different techniques to improve generalisation or eliminate overfitting of trained network. They use constrained minimization of training error. ‘trainlm’ uses ‘early stopping’; that is stop before convergence if error on validation subset starts increasing. This requires some training records (validation set) to be reserved in order to check overfitting. The ‘trainbr’ uses Bayesian regularization. It minimizes weighted sum of squared weights/ biases along with the training error to eliminate overfitting. ‘trainbr’ doesn’t require to reserve any records for validation. The results of ‘trainbr’ were found to be superior than the ‘trainlm’ and was therefore selected for further work. There are internal data pre and post processing functions provided by MATLAB. It is reported that ‘trainbr’ works well if input variables are normalized in the range [-1 + 1]. Appropriate parameters were set for this. The ‘divideFcn’ property of the network defines the data division function to be used when the network is trained using a supervised algorithm, such as backpropagation. This property was set to ‘Null’ so that there was no division of records; all the records used in the program were entirely for training. Taking value of $N_{\mathrm{Train}}$ as 178, Equation (1) was used to calculate $H_{\max}$ as 12. The highest number of neurons in the single layer model (#12) set the upper limit on the total neurons to be used while selecting multilayer model to 12. After preliminary numerical experiments, a ‘5–4-2′ structure was selected to be used for multiple layer ANN models. This structure satisfies the thumb rules mentioned in Section 2.1 and at the same time keeps the total neurons to $H_{\max}$. Similar ANN models were used for all the three variables of interest: jet velocity, •OH radical generation and energy dissipation rates. For the sake of brevity, single layer ANN models are not shown here. The architecture of the final multi-layer networks along with key parameters is shown in Fig. 2. The results obtained using these ANN models are discussed in the following section.

## 3 Results and discussion

ANN models for jet velocity, OH radical generation and localised energy dissipation rate (denoted by JV, OHG and TEDR respectively) were developed and tested following the steps outlined in Section 2.

### 3.1 Selection of appropriate ANN models

The best single layer model in the families of JV, OHG and TEDR was selected by constructing 1000 different networks for increasing values of neurons from 3 to 12 and choosing the one with a highest R-squared value. As mentioned in Section 2.1, R-squared is a measure of how close the predictions are to the actual values with 1 indicating a perfect match. Multilayer models were built with ‘5–4-2′ architecture with three hidden layers comprising 5, 4 and 2 neurons in that sequence. Similar to single layer models, 1000 different networks were constructed for multilayer models and the one with highest R-squared value was selected. Table 2 lists R-squared values for best single-hidden-layer and multiple-hidden-layer models for the three variables of interest. Number of neurons used in the model are indicated by ‘#H’. Total number of parameters (P_Total_) for every network which is a function of number of layers, their biases and number of neurons in each layer is also provided for comparison. Following discussion compares the two models (single/ multiple hidden layers) for each variable. They are referred as Model A and Model B for the ease of discussion.

• JV: it was observed that model A could provide R-squared value more than 0.995 for the highest number of neurons (12) for training set. Model B provided equal value for R-squared, but had considerably less number (#60) of total parameters as compared to Model A (#84). Increased number of parameters imply more complex model and chances of overfitting. Hence, Model B was selected for further evaluation.
• OHG: It is important to note that since the range of this variable was spanning 11 decades, the data was pre/ post-processed with logarithmic/ exponential functions before/ after training the network. Model A could reach the maximum R-squared value of 0.995 for the pre-processed variable. The Model B could reach the maximum R-squared value of 0.998 with lower number of total parameters. Therefore, Model B was selected for OHG as well.
• EDR: Model A failed to offer expected performance even at the highest possible number of neurons. The maximum value of R-squared value was 0.967 - less than the expected 0.995. Model B could give the value of R-squared as 0.997 with lower number of total parameters compared to Model A. Therefore, for the case of EDR as well, Model B was selected for further evaluation.

**Table 2:** R-squared values for ANN models.

ANN	Single hidden layer (model A)					Three (5–4-2) hidden layers (model B)				
name	#H	P_Total_	R^2^_Train_	R^2^_TestInt_	R^2^_TestExt_	#H	P_Total_	R^2^_Train_	R^2^_TestInt_	R^2^_TestExt_
JV	12	84	0.998	0.997	0.988	12	60	0.998	0.999	0.994
OHG	12	84	0.995*	0.985*	0.933*	12	60	0.998*	0.996*	0.988*
EDR	12	84	0.967*	0.985*	0.955*	12	60	0.997*	0.996*	0.940*

* based on natural log of corresponding variables.

**Fig. 3:**
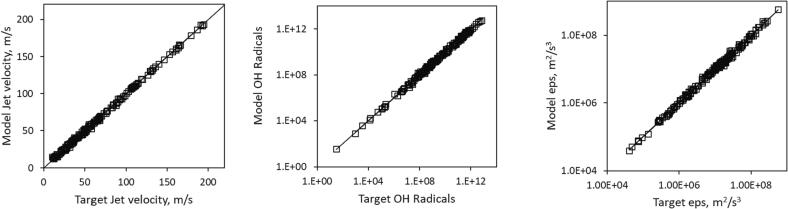
Parity plots for training model-B for all three variables.

Overall, Model B for all three variables showed higher R-squared values at lower number of total parameters. Fig. 3 shows the parity plots for training of type B models for the three variables.

Evaluation of the performance of both types of models was carried out by testing them on the two data sets that were not used while training the network models. The results are discussed in the following section.

### 3.2 Performance of network models for unseen data

Performance of identified best ANN models was evaluated separately for the two unseen data sets; one containing 23 records with value of $P_{A}^{\prime }$ equal to 2 and second containing 24 records with value of $P_{A}^{\prime }$ equal to 3. These particular values fall within/ out the range of records used while training. Hence testing the performance on these two groups separately offers the model behaviour regarding interpolation and extrapolation.

The ANN models were used to simulate three variables of interest for the unseen range of input parameters. The R-squared values of interpolation test, R^2^_TestInt_ and of extrapolation test, R^2^_TestExt_ are listed in the last two columns corresponding to Model A and Model B of Table 2. It can be seen that the R-squared values for Model B were higher than for Model A in each and every case. Further, these values were greater than 0.99 for all the three models when tested on interpolated data. Model JV offered value greater than 0.99 even for extrapolated data points. Model A for OHG gave rather low value of R-squared (0.933) for extrapolation. Model B for the same case showed much better performance with R-squared equal to 0.988. This value for OHG is satisfactory considering very high range of the variable. Overall, the performance of Model B (three hidden layers) was found to be very good even for extrapolation for all the three variables of interest.

**Fig. 4:**
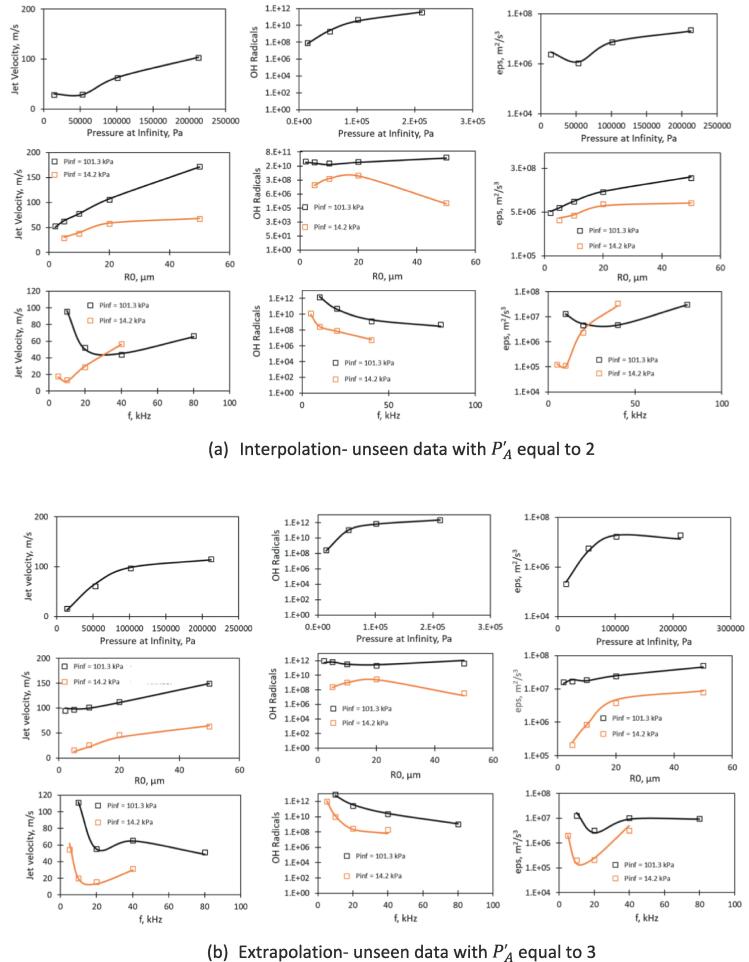
Dependency for variables as predicted by ANN models (continuous line) along with predictions using CDM (discrete points).

Rather than simply evaluating the overall quality based on R-squared value, it was also decided to closely examine influence of three key variables of cavity dynamics namely $P_{\infty }$, $R_{0}$ and f on jet velocity, hydroxyl radical generation and localised energy dissipation rate. The comparison of results simulated using the three layer dense ANN models developed in this work with those simulated using cavity dynamics model of Pandit et al. [4] is shown in Fig. 4a and 4b for interpolation and extrapolation respectively. As expected from the high values of R-squared in Table 2, the predictions of ANN models and the solutions of CDM match very well for all the cases. The complex dependencies of jet velocity and localised energy dissipation rate on $P_{\infty }$ and f are very well captured by the ANN models for both the test cases. It is really satisfying to see that the ANN model was able to interpolate as well as extrapolate all the three key variables of interest very well and have captured all the influences qualitatively as well as quantitatively very well. The model can therefore act as an excellent surrogate model for a detailed cavity dynamics model. The set of algebraic equations representing the best ANN models are presented in the following section.

### 3.3 Surrogate models

The ANN model may be represented by a set of algebraic equations involving input variables and various matrices associated with the trained network as:

(3) $$\mathrm{Output}=f^{4}\left({\mathrm{LW}}_{43}f^{3}\left({\mathrm{LW}}_{32}f^{2}\left({\mathrm{LW}}_{21}f^{1}\left({\mathrm{IW}}^{11}\times \left[G_{i}\left(P_{i}-O_{i}\right)-1\right]+b^{1}\right)\right)\right)\right)$$

Where P: R × 1 input vector where R is number of variables, i denotes element number.

G: R × 1 gain vector.

O: R × 1 offset vector.

$b^{1}$: S^1^ × 1 bias vector for layer 1.

S^1^..S^4^: number of neurons in layers1...4.

$f^{1}..f^{4}$: transfer functions for layers 1...4.

${\mathrm{IW}}^{11}$: S^1^ × R input weights matrix.

${\mathrm{LW}}_{\mathrm{ij}}$: weights matrix for layer number ‘i’ coming from layer number ‘j’.

The form of equation indicates cascaded layers where every layer gets a weighted input from previous output followed by a transfer function to generate its own output. Input vector with five elements as [P_∞_, f, R_0_, T_∞_, P_a_] is the same for all models. The values of matrices appearing in Equation (3) for each model are provided in three separate sheets of an Excel file in the supplementary information. ${\mathrm{IW}}^{11}$ is a 5 × 5 matrix which contains weights corresponding to 5 inputs, each going to every neuron in the first layer. Bias is only provided for the first hidden layer. Hence bias vector $b^{1}$ is a 5 × 1 matrix. The transfer functions for three hidden layers ($f^{1}..f^{3}$) is ‘Tansig’ which restricts the layer outputs within the range of −1 to 1. The layer weight matrices provide weights for the outputs from layers 1, 2 and 3. They have dimensions as 5 × 4, 4 × 2, 2 × 1 respectively considering the number of neurons in these layers. The transfer function of last layer was set to ‘Purelin’ which doesn’t modify the layer output in any way. Three surrogate model equations can be constructed by substituting appropriate matrices from supplementary information in Equation (3). It was verified that each of these algebraic equations provided the same output as given by the corresponding trained ANN in MATLAB.

Such availability of surrogate models has a potential to significantly enhance fidelity of representing physico-chemical effects of cavity collapse into device/ reactor scale models as discussed in Sarvothaman et al. [20] or Ranade [10]. The Equation (3) and corresponding matrices in the supplementary information provide the algebraic equations to estimate jet velocity, •OH generation and localised energy dissipation rates by cavity collapse and can be incorporated in any higher level models for simulating particle/ drop breakage, reactions of hydroxyl radicals for degradation of pollutants or any other cavitation based process applications. Estimation of jet velocity and corresponding hammer pressure may also be used for estimating extent of erosion. It may be noted that the estimates of the presented surrogate model capture physico-chemical effects of individual cavity collapse. Interaction among cavities within a cluster of cavities may influence resulting physico-chemical effects (see for example, Arora et al. [21], Yasui et al. [22]). The influence of neighbouring cavities is typically of second order. The presented model can capture the first order physico-chemical influence of collapsing cavity. Secondly, the approach presented here can be extended in a straightforward manner for developing the surrogate model based on dynamics of collapsing cavity cluster. We hope that the presented approach and the surrogate ANN model will stimulate development of high fidelity multi-scale modelling of cavitation based processes which may lead to step improvement in our ability of optimisation and scale-up of such applications.

## 4 Conclusions

ANN models were developed as surrogate models for rigorous cavity dynamics models. For minimising number of parameters involved in the ANN model, three separate models with single output were developed for simulating key physico-chemical effects of cavitation namely jet velocity, •OH generation and localised energy dissipation rate. Entire training data containing 225 records was available for each model due to this approach, leading to less overfitting. ANNs with three hidden layers with full connectivity were found to perform better than single hidden layer models. Due to cascaded stages, they could model the complex relation between the inputs and the output with lesser number of total parameters; again, leading to less overfitting or more generalisation. Thousand different ANNs were trained for each variable and the one showing highest value of R-squared was chosen. All the trained models had R-squared greater than 0.99. They offered excellent performance on unseen data both within and outside the training range. This indicates that the developed models were able to capture the underlying phenomena adequately well. ANN models were represented by set of algebraic equations for all the surrogate models and associated coefficient matrices for individual models are included in the supplementary information. The presented approach, models and algebraic equations will be useful for accurately representing physico-chemical effects of cavitation in higher order models for simulating cavitation based processes.

## CRediT authorship contribution statement

**Nanda V. Ranade:** Investigation, Data curation, Validation. **Vivek V. Ranade:** Conceptualization, Funding acquisition, Supervision.

## Declaration of Competing Interest

The authors declare that they have no known competing financial interests or personal relationships that could have appeared to influence the work reported in this paper.

## Data Availability

Data will be made available on request.
